# High Poverty and Hardship Financing Among Patients with Noncommunicable Diseases in Rural Haiti

**DOI:** 10.5334/gh.388

**Published:** 2020-02-06

**Authors:** Gene F. Kwan, Lily D. Yan, Benito D. Isaac, Kayleigh Bhangdia, Waking Jean-Baptiste, Densa Belony, Anirudh Gururaj, Louine Martineau, Serge Vertilus, Dufens Pierre-Louis, Darius L. Fenelon, Lisa R. Hirschhorn, Emelia J. Benjamin, Gene Bukhman

**Affiliations:** 1 Boston University School of Medicine, Boston, MA, US; 2 Boston Medical Center, Boston, MA, US; 3 Partners In Health, Boston, MA, US; 4 Harvard Medical School, Boston, MA, US; 5 Zanmi Lasante, HT; 6 Hôpital Universitaire de Mirebalais, HT; 7 Washington University in Saint Louis, MO, US; 8 Tufts New England Medical Center, Boston, MA, US; 9 Feinberg School of Medicine, Northwestern University, Chicago, IL, US; 10 Boston University School of Public Health, Boston, MA, US; 11 Brigham and Women’s Hospital, Boston, MA, US

**Keywords:** noncommunicable disease, global health, poverty, equity, hypertension, diabetes

## Abstract

**Background:**

Poverty is a major barrier to healthcare access in low-income countries. The degree of equitable access for noncommunicable disease (NCD) patients is not known in rural Haiti.

**Objectives:**

We evaluated the poverty distribution among patients receiving care in an NCD clinic in rural Haiti compared with the community and assessed associations of poverty with sex and distance from the health facility.

**Methods:**

We performed a cross-sectional study of patients with NCDs attending a public-sector health center in rural Haiti 2013–2016, and compared poverty among patients with poverty among a weighted community sample from the Haiti 2012 Demographic and Health Survey. We adapted the multidimensional poverty index: people deprived ≥44% of indicators are among the poorest billion people worldwide. We assessed hardship financing: borrowing money or selling belongings to pay for healthcare. We examined the association between facility distance and poverty adjusted for age and sex using linear regression.

**Results:**

Of 379 adults, 72% were women and the mean age was 52.5 years. 17.7% had hypertension, 19.3% had diabetes, 3.1% had heart failure, and 33.8% had multiple conditions. Among patients with available data, 197/296 (66.6%) experienced hardship financing. The proportions of people who are among the poorest billion people for women and men were similar (23.3% vs. 20.3%, p > 0.05). Fewer of the clinic patients were among the poorest billion people compared with the community (22.4% vs. 63.1%, p < 0.001). Patients who were most poor were more likely to live closer to the clinic (p = 0.002).

**Conclusion:**

Among patients with NCD conditions in rural Haiti, poverty and hardship financing are highly prevalent. However, clinic patients were less poor compared with the community population. These data suggest barriers to care access particularly affect the poorest. Socioeconomic data must be collected at health facilities and during community-level surveillance studies to monitor equitable healthcare access.

**Highlights:**

- Poverty and hardship financing are highly prevalent among NCD patients in rural Haiti.
- Patients attending clinic are less poor than expected from the community.
- People travelling farther to clinic are less poor.
- Socioeconomic data should be collected to monitor healthcare access equity.

## Introduction

Noncommunicable diseases (NCDs) such as hypertension, diabetes, and heart failure are major causes of morbidity and mortality in Haiti, and other low-income countries [1]. In the latest nationally-representative community-based survey, hypertension and diabetes were highly prevalent. Among adult women and men in Haiti, an estimated 49% and 38% have hypertension and 14% and 8% have diabetes, respectively [2]. Heart failure is a major cause of hospitalizations in urban and rural referral centers [3,4].

In Haiti, as in other low-income countries, care of chronic NCD conditions is accomplished primarily in health centers and hospitals, as opposed to community-based dispensaries [5]. However, there are significant barriers to chronic care access in rural Haiti [6,7,8,9]. Such barriers are more challenging for patients struggling with poverty and include long distances to health facilities, high out-of-pocket spending for clinical visits and transportation, and other competing priorities. The United Nations has adopted Sustainable Development Goal (SDG) 3 to promote healthy populations world-wide [10,11]. Specifically SDG 3.4 targets reducing premature mortality from NCDs, whereas SDG 3.8 aims to achieve universal health coverage and financial risk protection. Together, these two SDG targets are specifically relevant for people living with NCDs in rural, low-income country settings. To monitor progress on improving access to care for the poor, health systems must measure poverty among people who are and, ideally, are not able to obtain care.

Assessing poverty in low-income country clinic populations can be challenging. Resorting to proxy measures that may be collected during a typical patient registration process such as urban vs. rural address status may misclassify the poor. It is difficult to accurately measure income or health spending without administering detailed questionnaires [12]. Further, poverty encompasses many dimensions beyond income. Multidimensional metrics that incorporate living standards, education, and health may be more relevant among the poor [13].

The established key indicator to measure financial risk is to measure the proportion of total household expenditure that is related to health [11]. However, such indicators are often difficult to measure and may not fully assess the impact of health payments on consumption and poverty [14]. Hardship financing – borrowing money or selling assets to pay for unexpected medical costs – accounts for the source of household financing for unexpected medical costs [15,16]. Borrowing and selling may be more easily recalled by individuals, captures indirect spending such as transportation and lost income, and better reflects the detrimental impact of adverse coping strategies on families and households.

In this study, we aimed to first define the socioeconomic profile of patients presenting to an NCD clinic in rural Haiti using both multidimensional poverty and hardship financing. Then, we evaluated for equity in care access by comparing poverty distributions between the clinic patients and a community-based sample and evaluated for associations between distance to facility and poverty.

## Methods

### Design, Setting, and Subjects

This study was a cross-sectional study using two data sources. We studied patients with NCDs at Hôpital Universitaire de Mirebalais (HUM) enrolled in a clinical program from 2013–2016 to analyze their poverty intensity and evaluate associations between poverty and distance from the clinic. We then compared the poverty distribution of community members sampled during the 2012 Haiti Demographic Health Survey (DHS) with the NCD patients.

HUM is a 300-bed hospital and health center in the rural Central Plateau of Haiti. HUM is operated in partnership between the Haitian Ministry of Public and Population Health and the non-governmental organizations Zanmi Lasante (Haiti-based) and Partners In Health (Boston-based). The health center at HUM serves a primary care population of the 165,000 people who live closest to the hospital. However, patients throughout Haiti may self-present to the health facility of their choice for care. There is a one-time registration fee of about US$2 after which clinic visits, hospitalizations, diagnostic tests, and medications are provided for free.

A dedicated NCD clinic was established when HUM opened in March 2013. A general physician first evaluates patients in the outpatient clinic or hospital. Standard diagnostic and management protocols are used to identify patients with hypertension, diabetes, heart failure, and chronic respiratory diseases. They are then referred to the NCD clinic where their diagnosis is confirmed. Patients who agree to long-term follow-up were enrolled in the NCD clinic. We included all adult patients at least 18 years old who were enrolled in the HUM NCD clinic and had socioeconomic data recorded between March 2013 and October 2016. Data collection was occasionally incomplete. Thus, we excluded patients with missing data on all nine poverty indicators described below.

### Measurements and Data Collection

In the NCD Clinic at HUM, demographic and socioeconomic data are collected from patients at clinic enrollment and recorded on paper intake forms. These data include age, sex, education, occupation, health status of household members, transport, house assets, and hardship financing. Data on paper forms were manually extracted and entered into an electronic database by GFK and AG for the study. The electronic database was hosted on a secure and confidential web-based application: Research Electronic Data Capture (REDCap, Vanderbilt University, Nashville, USA) [17].

### Demographic Health Survey Data

The 2012 Haiti DHS is a nationally representative household survey using a 2-stage cluster sampling design conducted by the Haitian Ministry of Public Health [18]. A systematic sample of 13,277 households were selected within 445 selected clusters nationwide. All women in the sampled households aged 15 to 49 years (14,287), and all men aged 15 to 59 years (9,493) in two thirds of the households were interviewed. Data from health, education, and living standards categories are obtained from each household. Data are representative at the first sub-national level (department). Each cluster is georeferenced using displaced coordinates to preserve confidentiality [19]. The georeferenced dataset was spatially joined with a polygon shapefile of Haiti’s subnational regions.

To compare the poverty profile of NCD Clinic patients with the surrounding community, we developed a weighted community sample within the 2012 Haiti DHS. We weighted the DHS data using the NCD Clinic’s geographical distribution by Department to minimize differences in poverty due to differences between Departments. NCD clinic patients predominantly live in the *Artibonite, Centre* (Center), and *Ouest* (West) departments. The 2012 Haiti DHS subdivides the *Ouest* department into the *Aire Me?tropolitaine* (Metropolitan Area) around the capital Port-au-Prince, and non-metropolitan area.

### Poverty Assessment

We defined poverty using several measures.

#### Deprivation among poverty indicators

To define poverty, we adapted the multidimensional poverty index (MPI), a standardized poverty measure developed by the Oxford Poverty and Human Development Initiative and the United Nations Development Programme [13]. The MPI examines ten indicators among three dimensions commonly included in DHS samples worldwide: health (child mortality, nutrition), education (years of schooling, attendance), and living standards (cooking fuel, sanitation, water, electricity, floor material, and assets). Each indicator has pre-existing binary definitions for deprivation. We examined nine indicators as electricity availability was not assessed in the NCD clinic population. The standard MPI measure for nutrition is the presence of any person in the household who is underweight. We adapted the nutrition measure and categorized individual patients as underweight if their measured body mass index was <18.5 kg/m^2^. The poverty indicators and corresponding definitions of deprivation are summarized in supplement Table S1.

#### MPI

The MPI is a linear value (range 0–1) that can be calculated for different populations using two variables: 1) number of deprived indicators, and 2) whether household are considered MPI poor (i.e. deprived in ≥33% of all indicators). The MPI value for a population was calculated as the product of H × A, where H = number of households who were MPI poor/total population, and A = sum of weighted scores in indicators among households who were MPI poor/total MPI poor population [13]. MPI can only be calculated for participants with complete data on all poverty indicators. We calculated the MPI estimate and 95% confidence intervals for the subset of the NCD clinic population with complete socioeconomic data (n = 181). We also calculated the MPI estimate of for Haiti nationally and the weighted community sample using the 2012 DHS data, which has complete socioeconomic data.

#### Hardship financing

We assessed whether or not patients used different coping strategies to manage healthcare expenses. We asked patients if they previously (1) borrowed money, or (2) sold belongings to pay for healthcare [15,16].

#### Quintiles by indicator deprivation

The NCD clinic patients were categorized by wealth quintiles. The quintile ranges were determined by 2012 Haiti DHS percent deprived for Haiti nationally, and individually for each of the 1^st^ subnational departments where NCD patients live: *Centre, Artibonite, Ouest, and Aire Me?tropolitaine*. There should be equal frequencies of patients in each quintile if the NCD Clinic poverty distribution was the same as overall in Haiti and its departments.

### Analysis

#### Demographics and Poverty

We evaluated the demographic characteristics, diagnoses, and self-reported transportation time among men and women who attended the NCD Clinic. Differences were assessed using the Chi square test.

Multiple measures of poverty were calculated for the NCD Clinic population using clinic data, and for Haiti overall using DHS data. First, we assessed the percent of indicators deprived out of total collected indicators for each individual to avoid over- or underestimating poverty due to missing data. We compared the poverty distribution between the NCD Clinic population and the weighted community sample using Fisher’s exact test. People who are deprived in ≥4 of the 9 (≥44%) indicators are among the world’s poorest billion people [20,21]. Second, the poorest individuals within the NCD Clinic were identified as those whose percent of indicators deprived was greater than the mean [21]. Third, we compared the calculated MPI values for the NCD clinic and weighted community populations using the t-Test. Fourth, we generated poverty histograms by wealth quintile to compare NCD Clinic patients to the Haitian national poverty distribution, as well as for the subnational departments.

#### Sensitivity analyses

We performed several sensitivity analyses to assess the stability of our results given the incompleteness of the NCD clinic poverty data. The measure of percent of indicators deprived was calculated for the subset of patients with (1) complete socioeconomic (n = 181), and (2) at least one indicator with missing values imputed using the *mi impute* function in Stata (n = 335). After examining the number, proportion, and patterns of missing data, missing at random was assumed. We identified potential auxiliary variables, and performed imputation using multivariate normal regression, with 20 imputations (highest fraction of mission information 0.168). After imputation we checked for accuracy by examining the new mean values for each deprivation indicator. We also assessed the distribution of NCD clinic patients by wealth quintiles using the subset of patients with complete socioeconomic data.

#### Geospatial Analysis

To compare NCD Clinic patient poverty across geographic areas, administrative boundary shapefiles were downloaded from Humanitarian Data Exchange into ArcMap 10.6.1 (Esri, Redlands, CA). Clinic patients were mapped to their communal sections (third major subnational level). Within each communal section, we calculated the mean of the percent of socioeconomic indicators in which people were deprived.

We evaluated the relationship between poverty and distance to the clinic. We calculated the Euclidean distance from the geographic centroid of each communal section to HUM using the Near Distance Spatial Analyst tool in ArcMap. The relationship between distance from clinic (independent variable) and percent of deprived indicators (dependent variable) was determined using Spearman’s correlation and simple linear regression with robust standard errors adjusting for age and sex.

### Research Ethics

This study was approved by the Zanmi Lasante Ethics Committee in Haiti and the Boston University Medical Campus Institutional Review Board. As the study involved analysis of de-identified routinely collected clinical data, individual consent was waived. All analyses were performed in Stata/SE 13.1 (StataCorp, College Station, TX). P-values <0.05 were considered statistically significant.

## Results

A total of 515 adults were enrolled in the NCD clinic during the study period. 379 adults (73.5%) had data recorded on at least one poverty indicator and were included in the analytic cohort. Shown in Table 1, 71.5% were women and the mean age was 52.5 years (52.3 years women, 53.0 years men). For comparison, among the weighted community sample, 48.6% (95% CI, 47.9% to 49.2%) are women. A large proportion of patients traveled less than 30 minutes to reach the clinic (43.0% of women, 36.4% for men), and no one traveled more than 6 hours. For medical conditions, 17.7% of patients had hypertension only, 19.3% had diabetes only, 3.1% had heart failure only, and 33.8% of people had more than one of these conditions. The demographic summary of the 181 patients who had complete data for all poverty indicators is similar to the analytic cohort (Supplementary Table S2).

**Table 1:** Demographics.

	Total	%	Women	%	Men	%	Poorest (<44% deprived)	%	Not poorest (<44% deprived)	%

N	379		271	(71.5%)	108	(28.5%)	85	(22.4%)	294	(77.6%)
Age, mean, y (sd)	52.5	(0.8)	52.3	(0.9)	53.0	(1.5)	54.3	(1.8)	52.0	(0.8)
Women	271	(71.5%)	–	–	–	–	63	(74.1%)	208	(70.7%)
Poorest (≥44% deprived)	85	(22.4%)	63	(23.2%)	22	(20.4%)	–	–	–	–
Body mass index, mean, kg/m^2^ (sd)	26.9	(0.4)	27.3	(0.5)	25.9	(0.8)	23.2	(0.9)	28.2	(0.5)
Transport duration										
<30 min	128	(41.2%)	96	(43.0%)	32	(36.4%)	42	(35.3%)	98	(33.3%)
30 min–1h	64	(20.6%)	42	(18.8%)	22	(25.0%)	17	(29.4%)	39	(13.3%)
1h–2h	76	(24.4%)	56	(25.1%)	20	(22.7%)	28	(12.9%)	65	(22.1%)
2h–3h	32	(10.3%)	21	(9.4%)	11	(12.5%)	11	(7.1%)	26	(8.8%)
3h–6h	7	(2.3%)	5	(2.2%)	2	(2.3%)	02	(2.4%)	5	(1.7%)
Don’t know	4	(1.3%)	3	(1.3%)	1	(1.1%)	0	(3.5%)	1	(0.3%)
Condition										
Hypertension only	67	(17.7%)	52	(19.2%)	15	(13.9%)	24	(28.2%)	43	(14.6%)
Diabetes only	73	(19.3%)	45	(16.6%)	28	(25.9%)	15	(17.6%)	58	(19.7%)
Heart Failure only	12	(3.2%)	8	(3.0%)	4	(3.7%)	4	(4.7%)	8	(2.7%)
Multiple	128	(33.8%)	95	(35.1%)	33	(30.6%)	22	(25.9%)	106	(36.1%)
Other	21	(5.5%)	17	(6.3%)	4	(3.7%)	4	(4.7%)	17	(5.8%)

### Poverty deprivations

**Figure 1:**
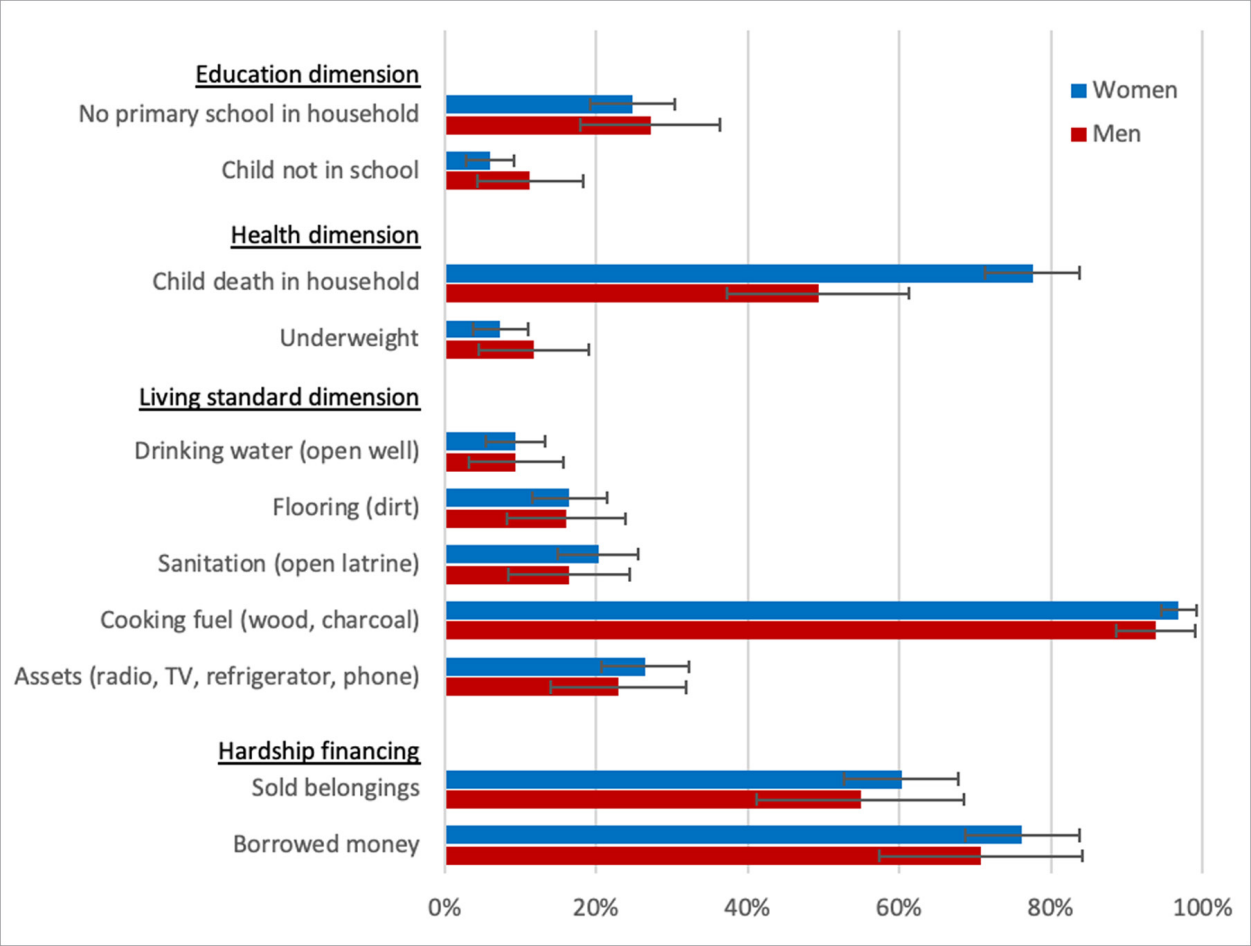
Percent of NCD Clinic patients deprived in each indicator for women and men, unadjusted. 95% Confidence intervals shown.

Among all participants, 307/379 (81.0%), had at least six of nine socioeconomic indicators available (Supplementary table S3). Among the nine deprivation indicators, the percent of people deprived was highest for cooking fuel (96.0%, 95% confidence interval [CI]: 93.8% to 98.2%), child death (69.5%, 95% CI: 63.7% to 75.4%), and years in school (25.6%, 95% CI: 20.1% to 30.4%). The proportion of NCD clinic patients by sex who were deprived in the nine poverty indicators is shown in Figure 1. Women reported more deprivation in child death than men (77.6% vs. 49.3%), while more men reported less deprivation in school age children not attending school (6.0% vs. 11.3%) and deprivation in body mass index (7.3% vs. 11.7%) (Table 2). Deprivations by indicator among patients who had complete poverty indicator data and for all patients with missing data imputed were similar to the analytic cohort (Supplement Table S4).

We calculated the percent of collected indicators in which a person was deprived, referred to as percent deprived. 23.3% (95% CI: 18.2% to 28.3%) of women and 20.3% (95% CI: 12.7% to 28.0%) of men were deprived in ≥44% of indicators and are among the world’s poorest billion (poorest 13%) people. Men (23.9%) and women (27.9%) were similar in percent deprived. The distribution of frequencies across percent deprived levels was different between the sexes, as shown in Figure 2A (p = 0.002). The calculated MPI among women (0.197, 95% CI: 0.154 to 0.239) was not statistically significant different than men (0.217, 95% CI: 0.132 to 0.303).

**Table 2:** Multidimensional poverty index among NCD clinic and community population.

Population		MPI (95% CI)^#^

Noncommunicable Disease Clinic*	Total	0.202 (0.164, 0.240)
	Women	0.197 (0.154, 0.239)
	Men	0.217 (0.132, 0.303)
Haiti 2012 Demographic and Health Survey	Community (weighted)	0.277 (0.247, 0.307)
	Haiti national	0.229 (0.215, 0.243)
	Urban	0.129 (0.113, 0.145)
	Rural	0.296 (0.275, 0.317)

^#^ MPI, Multidimensional Poverty Index (higher = poorer). * Patients with complete poverty data (n = 181).

**Figure 2A:**
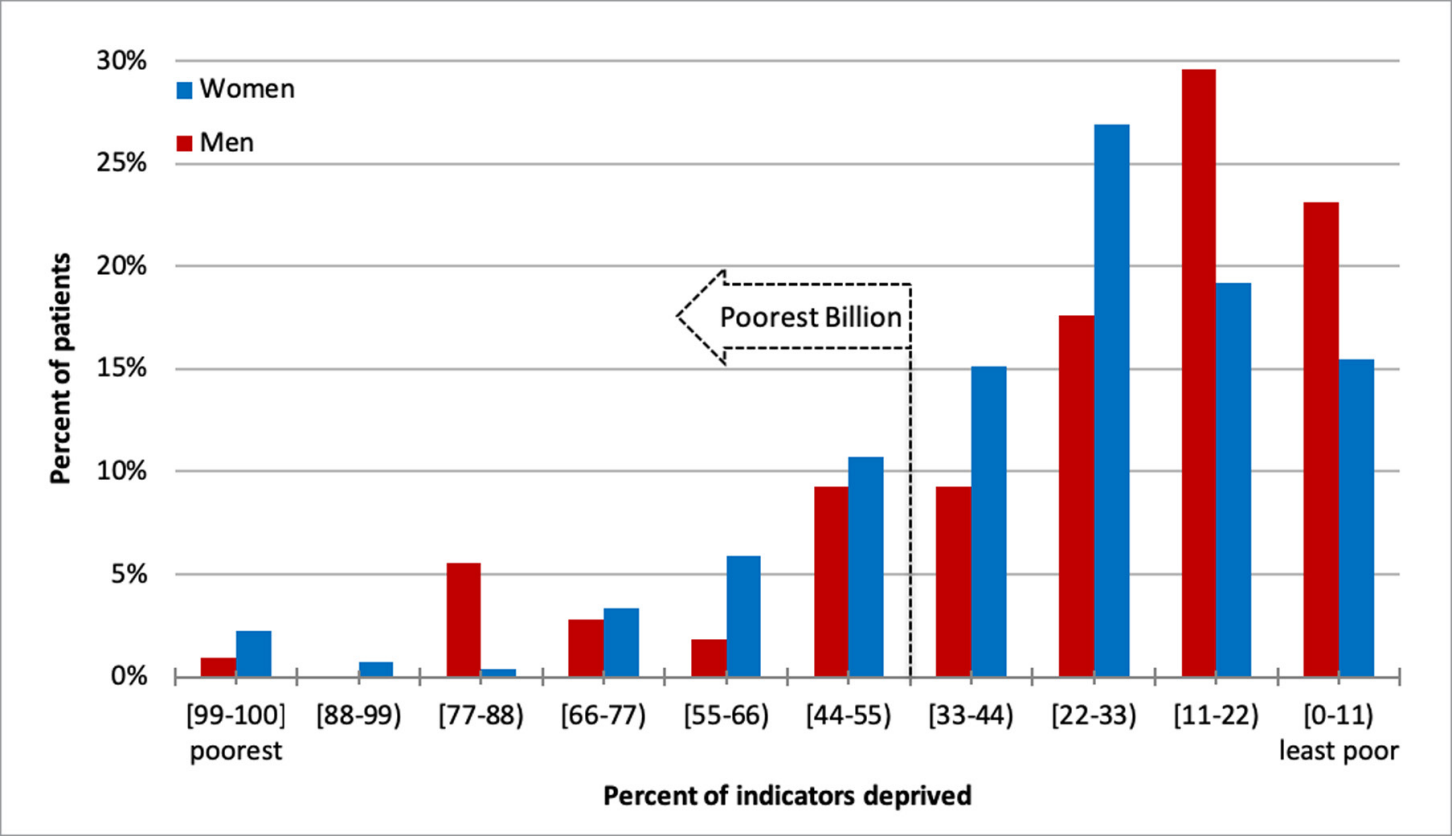
Proportion by deprivation status by sex among people in NCD Clinic, unadjusted. The proportion of people across deprivation levels is different between sexes (p = .002).

We also examined poverty using hardship financing. Of those with available data, 197/296 (66.6%) experienced any type of hardship financing: 148/214 (59.8%) for women vs. 49/82 (69.2%) for men. Among the clinic patients, 146/296 (49.3%) sold belongings and 178/292 (61.0%) borrowed money to pay for healthcare.

### NCD Clinic vs. Weighted Community Sample in Haiti

The NCD Clinic patients included in this analysis have lower deprivation scores than the surrounding weighted community sample. Figure 2B shows the percent deprived indicators between the NCD Clinic and the weighted community sample. NCD Clinic patients were overall less poor compared with the community (p < 0.001). The proportion of patients among the NCD Clinic who were among the poorest billion is smaller than for the weighted community sample (22.4% vs. 63.1%, p < 0.001). We calculated the MPI among the 181 NCD clinic patients with complete poverty data, shown in Table 2. In individuals with complete data the calculated MPI of 0.202 (95% CI 0.164 to 0.240) was lower than the weighted community’s MPI of 0.277 (95% CI 0.247 to 0.307) (p < 0.05).

**Figure 2B:**
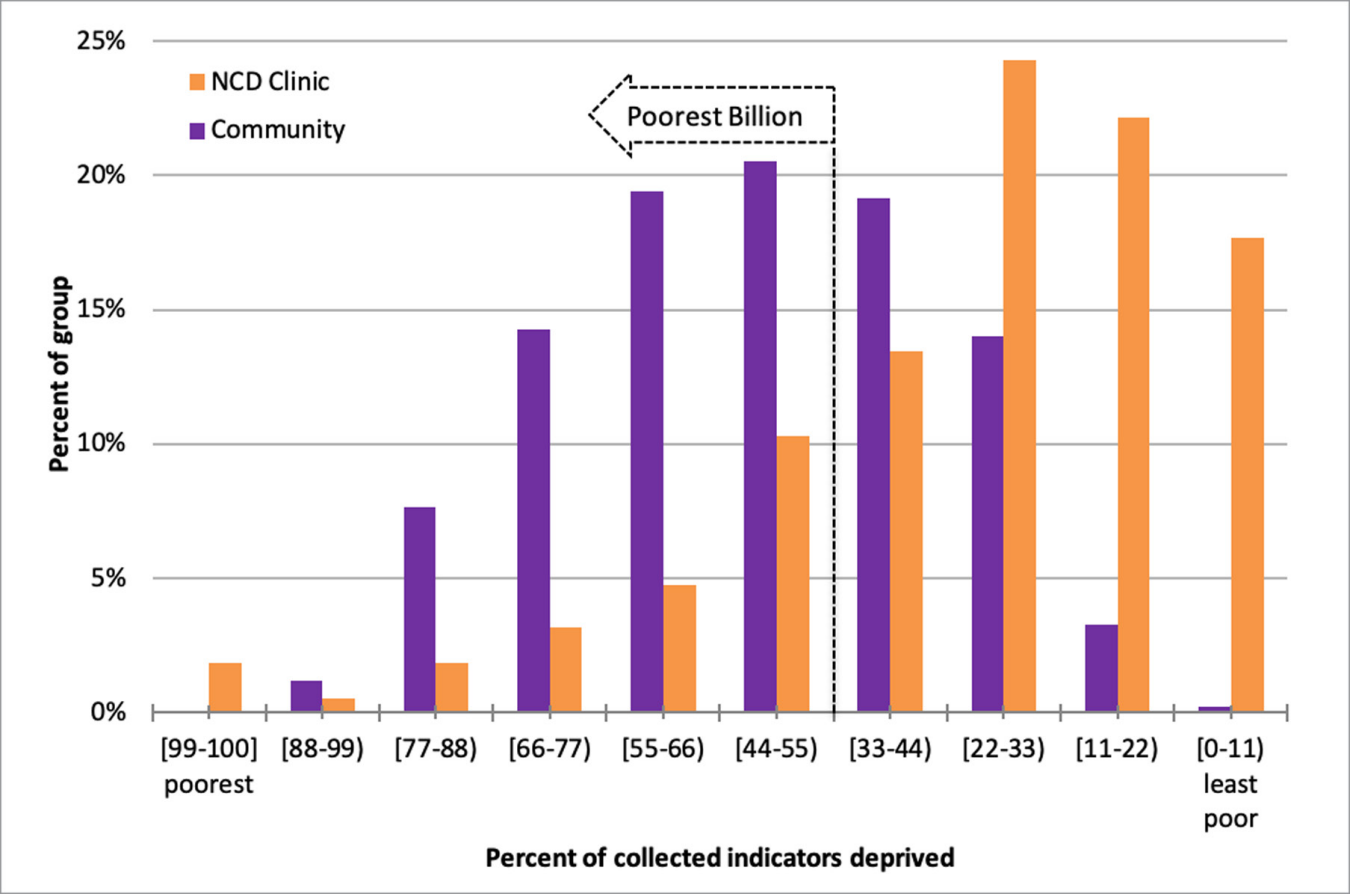
Poverty distribution among NCD Clinic vs. Community. Proportion of people by deprivation status, NCD Clinic (n = 378, 1 patient with missing address) vs weighted community sample, unadjusted. Poverty distribution is different between the NCD Clinic and Community groups (p < 0.001).

**Figure 3:**
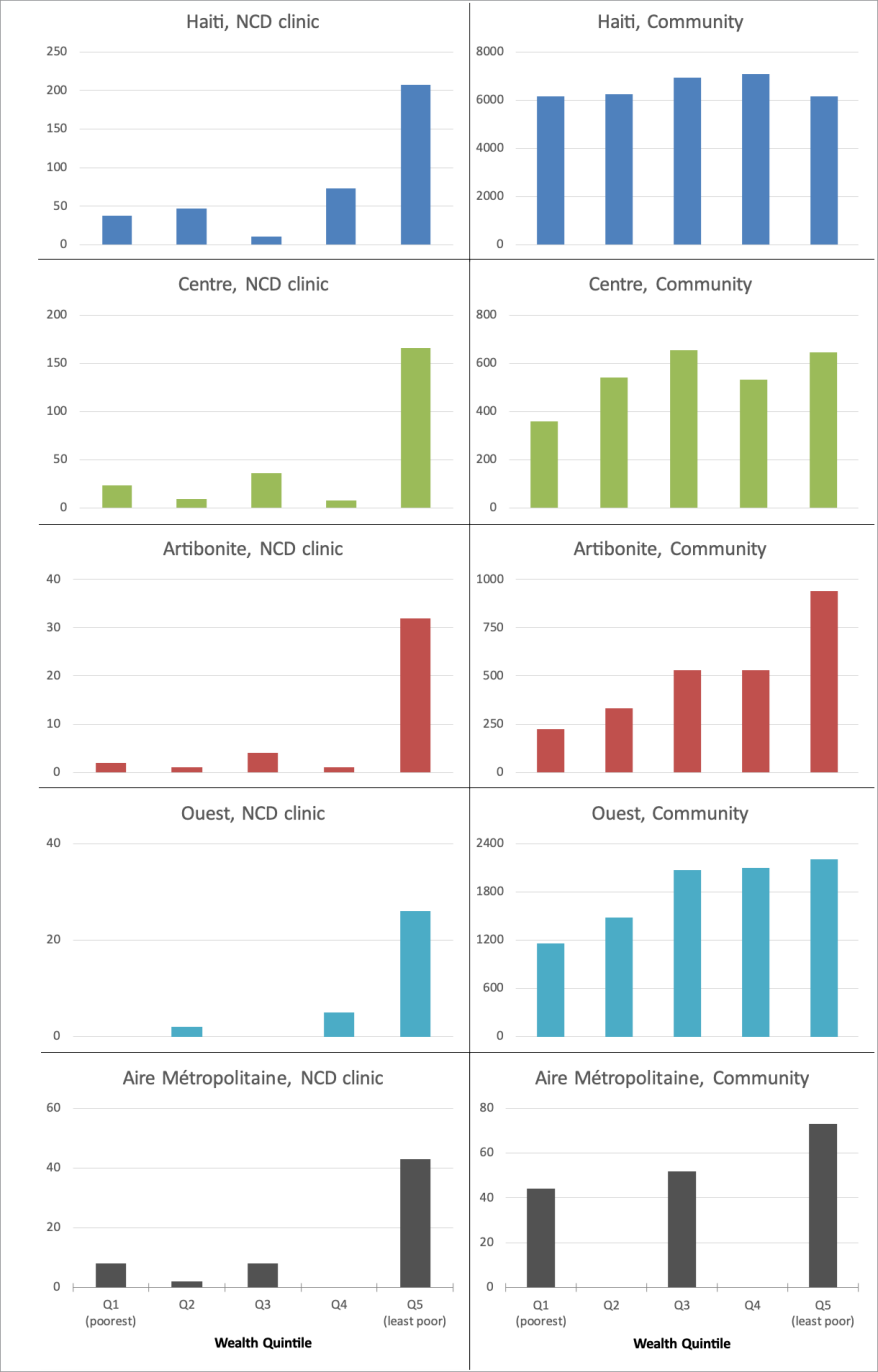
Number of people by wealth quintile in the NCD Clinic and Community nationally, and by geographic region. Wealth quintiles are defined using 2012 Haiti DHS data nationally, and individually for the major departments where NCD Clinic patients live.

We examined the distribution of NCD Clinic patients and the community by wealth quintiles, shown in Figure 3. Within the NCD Clinic, the majority of patients fell within the highest wealth quintiles nationally and within each Department. This distribution contrasts with the relatively equal proportion of community population in each wealth quintile. Supplementary Figure 2 shows the histogram for the subset with NCD Clinic patients with complete poverty indicator data.

### Geographical Distribution of Poverty

**Figure 4:**
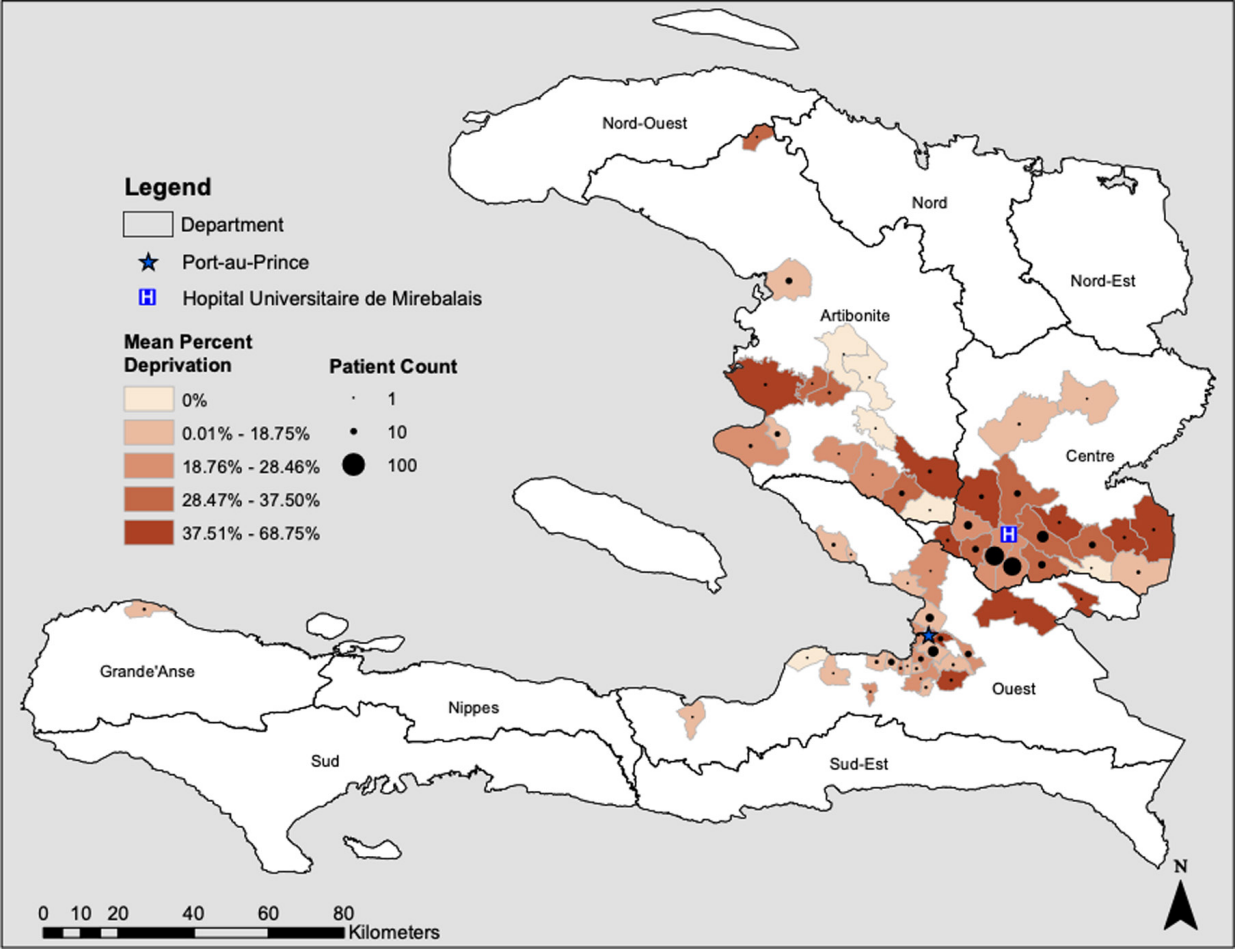
Map of number of NCD Clinic patients and poverty by communal section. The average percent deprivations for patients is shown in red shades. The number of patients is shown by the size of the circles.

Patients who lived closer to the clinic were poorer. The map in Figure 4 illustrates the geographic number and poverty (average percent deprivation) of patients by communal section – the 3^rd^ subnational level. Increasing poverty levels were associated with patients living closer to clinic (Spearman’s rho –0.16, p = 0.002 for correlation between distance from facility and poverty). In univariable linear regression, a one additional unit of poverty deprivation was associated with a 63 km decrease in distance. Sensitivity analyses with: 1) only people with complete data revealed a one unit increase in poverty level was associated with a 81 km decrease in distance and; 2) multiply imputed data showed a one level increase in poverty was associated with a 52 km decrease in distance. In multivariable linear regression adjusting for age and sex, a one unit increase in poverty level was associated with an 68 km increase in distance (p = 0.002) – shown in Supplement table S5.

## Discussion

Our study shows that multidimensional poverty and hardship financing are highly prevalent among patients in an NCD clinic in rural Haiti. However, the poverty distribution of patients receiving care in the clinic was heavily skewed away from the poorest compared with the communities where they live. This observed socioeconomic difference may be, in part, due to substantial barriers that inhibit the poorest patients from coming to clinic – such as far distance. To our knowledge, our study is the first to compare patient-level and community poverty using a multidimensional measure in Haiti. Achieving universal health coverage for NCDs in Haiti will require further investigation of poverty-related barriers and responsive health system adaptations to lower those barriers.

The NCD clinic patients in our study have a high prevalence of deprivation among individual poverty measures, and in multidimensional poverty. Nearly all patients use wood or charcoal for cooking, and most have suffered through the recent death of a child in the household. Further, despite heavily subsidized clinic-based care, about half of patients experienced hardship financing – indicating the detrimental impact of health spending on households. Overall, the deprivations are similar between men and women – with the exception of household child deaths.

However, compared with the estimates from the community, the clinic patients are less poor. Clinic patients are deprived in fewer poverty indicators, are less likely to be in lower socioeconomic quintiles, and have a lower MPI. Our findings were robust when evaluating poverty using multiple methods and in sensitivity analyses. Our observed difference in poverty profile among patients in a rural NCD clinic compared with the community could be explained by multiple factors including (1) lower health facility utilization among the poor – particularly those who live far from the facility, (2) lower prevalence of NCD conditions among the poor, and/or (3) variation in diagnosis of NCD conditions by income status (ascertainment bias). The prevalence of hypertension in Haiti has been reported to be higher among those who are most poor compared with those who are least poor [2]. This social gradient is reversed for diabetes and likely reflects lower obesity among the most poor compared with the wealthiest in the 2016–17 Haiti DHS [2] and among our patients.

Our geospatial analysis shows that NCD clinic patients who live closer to the health facility are generally poorer compared with those who live farther away, adjusted for age and sex. Importantly, our study included only patients who were able to present to the clinic. We were not able to assess people who did not come to the health facility and may experience higher barriers to care access including higher poverty or living farther away. It is very likely that the people in the community with NCD conditions living far away may be similarly very poor but are not able to access the clinic. Community-level data in Haiti show that higher poverty is closely associated with farther distances to the health facility. In the 2016–17 Haiti DHS, poor people tended to live farther from health facilities: 41% vs. 24% of the most and least poor, respectively, live more than 15 km from the nearest health facility [2].

Living farther from a facility can reduce access to care. A study of patients with heart failure at HUM showed lower adherence to follow-up care among those who lived farther away [3]. In a country where 36% of healthcare expenditure is out-of-pocket [22] additional transport costs can greatly influence access to NCD care. Further, higher poverty is also associated with lower awareness, treatment, and control of NCDs in Haiti [2], and in other low-income countries [23]. It is possible that patients who are poor and live far from HUM may seek care at facilities closer to their home or have lower health literacy and may not realize the importance of follow-up.

In our study, about three quarters of patients in the clinic were women. The pattern of having more women than men is similar to other clinic- and hospital-based data in Haiti [3,4,24,25]. This gender difference may be partially due to a higher prevalence of NCD conditions among women. The estimated prevalence among adults is higher for women than men for both hypertension (49% vs. 38%) and diabetes (14% vs. 8.2%) from the 2016–17 Haiti DHS [2]. and other low-income countries [26,27]. Also, there may be different barriers to care-seeking for men compared with women that warrant further study. Women may be more sensitized to seeking care at a health facility based on experiences with obstetric care. Among people with hypertension in Bangladesh, women tend to be more adherent to medications [28]. A study of patients with heart failure exacerbation in the United States showed that men had longer pre-hospital delays to care than women [29]. Gender differences in facilitators and barriers to care deserves further study to promote equity in healthcare access.

More intense poverty is associated with higher barriers to health facility utilization. These barriers to health-seeking behavior in rural Haiti must be evaluated and addressed to promote equitable access to NCD care. Chronic disease patients navigate multiple social adversities and competing priorities to meet basic needs [30]. In Haiti, there is a rich history of studies documenting the barriers to chronic care for human immunodeficiency virus. Patient factors include limited time to attend clinic, low income, food insecurity, poor social support, and cultural norms [31,32,33]. Environmental factors include transport costs [34], lack of healthcare facilities close to patients, road conditions, seasonal weather, and political disruptions. Health system factors include negative experience at hospitals, medicine costs [35], and complex systems [36]. Further research is needed to specifically identify the barriers to care for patients with NCDs in rural Haiti.

Interventions in Haiti to improve care access among the poor must specifically address identified barriers. In Haiti, health system interventions for the care of patients with human immunodeficiency virus have included food packages [6], community health worker-based adherence support [7,8], and improvements in clinic efficiency to shorten wait times and improve the care experience [9]. Health system interventions incorporating task shifting and task sharing to decentralize care towards smaller health facilities and communities that are closer to the people can reduce barriers to care access [37]. In Tibet and India, a mobile technology-supported non-physician health worker program improved hypertension control [38]. In Rwanda, a decentralized and nurse-led heart failure care program had good survival rates [39]. The emergence of mobile technologies and text message reminders may improve communication between patients and health providers [40].

Outside of the health sector, policies are necessary to address underlying poverty. Though we identified a high prevalence of deprivations among our patient cohort, improving living standards and access to education will require interventions largely influenced by Ministries of Education, Commerce, or Finance, and not only the Ministry of Health. Improving population health is a compelling argument to promote multi-sectoral interventions [41].

Within the health sector, improving equitable health service coverage requires disaggregation of health facility use information by socioeconomic status. Measures of poverty or hardship financing can be simple and informative, and must be incorporated into routine data collection at health facilities and in regular reporting. Assessing monetary poverty is often an unreliable and inaccurate measure of well-being in low-income countries [42]. Further, measuring spending alone does not capture the long-term detrimental impact of difficult coping strategies including borrowing money with high interest rates, removing children from school, reducing expenses on food and education, or quitting work to give care [43]. Traditional spending measures may underestimate the impact on families as they adjust spending in response to medical financial shocks [14,15]. While address data – classified as urban vs. rural status or using the specific geographic point – may be used as a proxy for individual poverty, we have shown that there is marked heterogeneity even among people in rural Haiti.

Multidimensional measures are a more holistic approach to poverty assessment. The 10 MPI indicators requires about 22 survey questions and is standardized across countries. The Equity Tool is tailored to each country and has 13 survey questions in Haiti [44]. More simple still is assessing hardship financing using only two questions. Health systems will need to balance socioeconomic instrument complexity, calibration, and feasibility.

Our cross-sectional study has several limitations. First, the collection of socioeconomic data of patients was inconsistent. Poverty assessment was highly dependent on clinic staffing and performed intermittently within the activities of a high-volume clinic. About a quarter of NCD clinic patients did not have socioeconomic data recorded. Thus, there may be selection bias towards patients considered to be more engaged in care, more adherent, and less poor. Second, the full set of socioeconomic indicators was not collected on each study participant. We assessed the robustness of our results through several sensitivity analyses among the subset of patients with complete socioeconomic data, and by using multiple imputation. When employing multiple imputation, we assumed that data were missing at random. However, missing data in our sample was likely not random. In our various sensitivity analyses, there was no meaningful change in our results among different subsets of patients. Third, our study was conducted at a single outpatient clinic associated with an academic referral hospital in rural Haiti. Our study site is unique, as user fees are very low compared with other health facilities in Haiti. While significant transportation and opportunity costs remain, patients presenting to HUM will likely be skewed towards the poorest given the very low out-of-pocket expense for healthcare visits. Fourth, the 2012 Haiti DHS sampling period (2012) did not overlap with the NCD clinic group (2013–2016). The prevalence of NCD conditions among residents in the community is not known for participants in the 2012 Haiti DHS. The DHS in Haiti is repeated every 4–5 years and the next was conducted in 2016–2017. Further, the DHS sampled women (15–49 years) and men (15–59 years) of different age ranges. When using the weighted community sample to the NCD clinic population, we did not standardize for age. Fifth, we cannot assess temporality or causality in our cross-sectional observational study. Sixth, we estimated distance using the Euclidean straight-line method. While our measure of distance did not account for different walking paths, roads directions, road quality, or transportation cost, it is a reasonable proxy for measuring spatial access [45].

## Conclusion

In our study among patients with NCD conditions in rural Haiti, we found a high prevalence of poverty and hardship financing. However, patients presenting for clinic-based care were less poor than would be expected compared with their communities. Further, patients travelling farther to the clinic tended to be less poor. These data indicate that poverty-related barriers may be substantial, deserve further study, and require targeted interventions. Individual-level socioeconomic data must be collected among patients at health facilities and during individual-level disease surveillance studies in the community to facilitate monitoring of equitable healthcare coverage. Without assessing poverty when planning and evaluating health system interventions, the poorest are at risk of being left further behind.

## Additional File

The additional file for this article can be found as follows:

10.5334/gh.388.s1 Supplemental Material. Adapted multidimensional poverty index indicators, and supplementary results tables and figures.
