# Supplementary material for: High Poverty and Hardship Financing Among Patients with Noncommunicable Diseases in Rural Haiti
Source: Glob Heart. 2020 Feb 6;15(1):7. doi: 10.5334/gh.388 (PMC7218772; doi:10.5334/gh.388)

Supplemental Material

Title: High poverty and hardship financing among patients with noncommunicable diseases in rural Haiti.

Authors:
Gene F. Kwan, MD MPH; Lily D. Yan, MD; Benito D. Isaac: Kayleigh Bhangdia; Waking Jean-Baptiste, MD; Densa Belony; Anirudh Gururaj; Louine Martineau, MD; Darius Fenelon, MD MPH; Lisa R. Hirschhorn; Emelia J. Benjamin, MD ScM; Gene Bukhman, MD PhD

Supplement Contents

Table S1	Page 2	Adapted Multidimensional Poverty Index
Table S2	Page 3	Demographics of patients with complete data on all poverty indicators (n=181).
Table S3	Page 4	Number people with available socioeconomic indicators.
Table S4	Page 5	Deprivations by poverty indicator among patients with complete
Table S5	Page 6	Multivariable linear regression model for associations with percent deprivations.
Figure S1	Page 7	Number of people by wealth quintile in the NCD Clinic and Community nationally, and by geographic region – among patients with complete data on all poverty indicators (n=181).

Table S1. Adapted Multidimensional Poverty Index

**Dimension of poverty**	**Indicator**	**Deprived if…**	**Adaptation from OPHI definition**
Education	Years of schooling	No household member has completed 6 years of schooling	Patient or head of household has not completed 6 years of schooling
Education	Child school attendance	Any school aged child is not attending school up to class 8	Any school aged child is not attending school
Health	Child mortality	Any child died in family in last 5 years	Any child died
Health	Nutrition	Any adult or child for whom there is nutritional information is stunted	Patient BMI < 18.5 kg/m2
Standard of living	Sanitation	Household sanitation facility is not improved, or is shared with other households	Household toilet is not improved (open latrine, bush)
Standard of living	Drinking water	Household does not have access to safe drinking water	Household does not use improved water source (anything other than tap, wells, protected water source, rain, commercial water)
Standard of living	Floor	Household has dirt, sand, or dung floor	Household has dirt floor
Standard of living	Cooking fuel	Household cooks with dung, wood, or charcoal	Household cooks with wood or charcoal
Standard of living	Assess ownership	Household doesn't have more than one of: radio, TV, telephone, bike, motorbike, refrigerator, and does not own car or truck	Household doesn't have more than one of: radio, TV, telephone, refrigerator

Note: Electricity was excluded for the poverty assessment used in this study.

Table S2. Demographics of patients with complete data on all poverty indicators (n=181).

	**Total**	**%**	**Women**	**%**	**Men**	**%**
N	181		135	(74.6%)	46	(25.4%)
Age, mean, y (sd)	55.1	(1.1)	54.2	(1.3)	57.7	(2.2)
Transport duration						
<30 min	72	(40.7%)	55	(41.7%)	17	(37.8%)
30min-1h	39	(22.0%)	28	(21.2%)	11	(24.4%)
1h-2h	41	(23.2%)	30	(22.7%)	11	(24.4%)
2h-3h	19	(10.7%)	13	(9.85%)	6	(13.3%)
3h-6h	3	(1.69%)	3	(2.27%)	0	(0%)
Don’t know	0	(0%)	0	(0%)	0	(0%)
Condition						
Hypertension only	45	(24.9%)	36	(26.7%)	9	(19.6%)
Diabetes only	41	(22.7%)	28	(20.7%)	13	(28.3%)
Heart Failure only	4	(2.21%)	3	(2.22%)	1	(2.17%)
Multiple	58	(32.0%)	44	(32.6%)	14	(30.4%)
Other	7	(3.87%)	5	(3.70%)	2	(4.35%)

Table S3. Number people with available socioeconomic indicators.

Number of socioeconomic indicators available	Number	Percent
1	54	14.3%
2	7	1.9%
3	1	0.3%
4	4	1.1%
5	6	1.6%
6	14	3.7%
7	41	10.8%
8	71	18.7%
9	181	47.8%
Total	379	100%

Table S4. Deprivations by poverty indicator among patients with complete data on all poverty indicators (n=181) and patients with missing data imputed (n=335).

Indicator	Patients with Complete Data (N=181)			Multiple imputation (N=335)		
	Percent %	95% Confidence Interval		Percent %	95% Confidence Interval	
Years of schooling	**34.8**	27.8	41.8	**31.5**	25.5	37.5
Child school attendance	**6.6**	3.0	10.3	**7.0**	3.6	10.4
household child deaths	**72.4**	65.8	79	**68.4**	62.1	74.8
BMI (Body Mass Index) ≥ 18.5%	**6.6**	3.0	10.3	**6.9**	3.4	10.3
drinking water	**11.6**	6.9	16.3	**10.8**	6.8	14.9
flooring	**19.3**	13.5	25.1	**17.4**	12.4	22.3
sanitation	**20.4**	14.5	26.4	**20.7**	15.5	26
cooking fuel	**96.1**	93.3	99	**94.6**	91.7	97.5
assets: radio, tv, refrigerator, phone	**25.4**	19.0	31.8	**27.7**	21.9	33.5
1 or more deprivations	**98.9**	97.4	100.4	**92.5**	89.6	95.4
2 or more deprivations	**82.3**	76.7	87.9	**68.9**	63.8	73.9
3 or more deprivations	**50.3**	42.9	57.6	**41.9**	36.4	47.4
4 or more deprivations	**29.3**	22.6	36	**25.0**	20.3	29.8
5 or more deprivations	**14.9**	9.7	20.2	**13.1**	9.4	16.8
6 or more deprivations	**10.5**	6.0	15.0	**7.5**	4.5	10.5
7 or more deprivations	**5.0**	1.8	8.2	**3.0**	1.2	4.9
8 or more deprivations	**1.7**	-0.2	3.5	**0.9**	-0.1	1.9
9 or more deprivations	**0.6**	-0.5	1.6	**0.3**	-0.3	0.9

Table S5. Multivariable linear regression model for associations with percent deprivations. Beta coefficients show the relationship between the variables and an increase in one level of poverty (increase by 11% of percent deprived indicators)

Variable	Beta	95% CI lower	95% CI upper	P value
Age (years)	0.0131783	-0.0024913	0.0288478	0.10
Sex (referent = men)	0.3909103	-0.0641988	0.8460194	0.09
Distance (km)	-0.0146585	-0.0219547	-0.0073625	<0.001
Intercept	1.788112	0.7909698	2.785254	<0.001

Figure S1. Number of people by wealth quintile in the NCD Clinic and Community nationally, and by geographic region – among patients with complete data on all poverty indicators (n=181). Wealth quintiles are defined using 2012 Haiti DHS data nationally, and for the major departments from which NCD Clinic patients live.

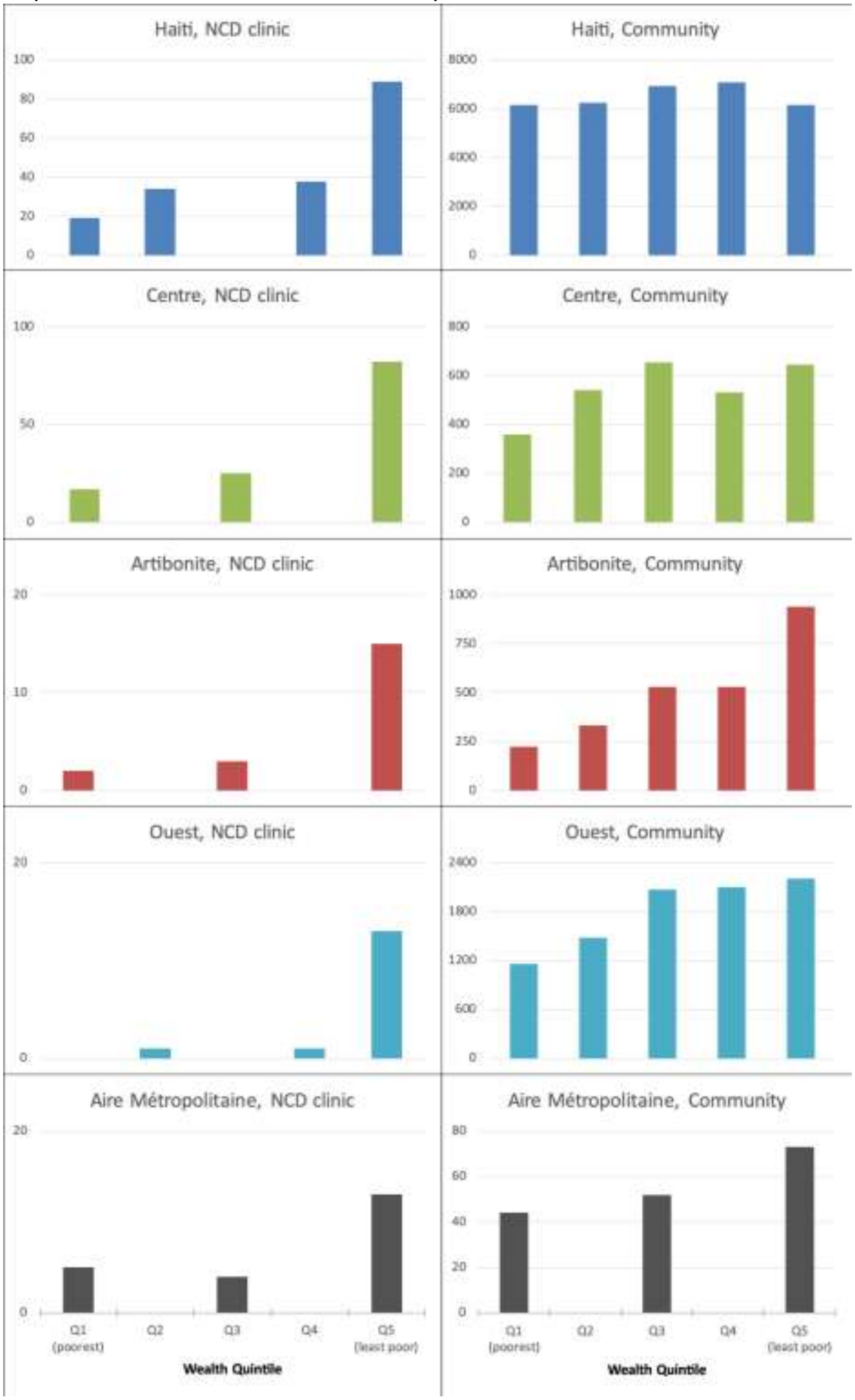

Supplement: Supplemental Material. — Adapted multidimensional poverty index indicators, and supplementary results tables and figures. [file gh-15-1-388-s1.pdf]
